# Clarifying the association between Parkinson’s disease and vitiligo: a population-based large-scale study

**DOI:** 10.3389/fneur.2024.1387404

**Published:** 2024-05-21

**Authors:** Khalaf Kridin, Lior Ofir, Orly Weinstein, Samih Badarny

**Affiliations:** ^1^Azrieli Faculty of Medicine, Bar-Ilan University, Safed, Israel; ^2^Clalit Health Services, Tel-Aviv, Israel; ^3^Unit of Dermatology and Skin Research Laboratory, Galilee Medical Center, Nahariya, Israel; ^4^Lübeck Institute of Experimental Dermatology, University of Lübeck, Lübeck, Germany; ^5^Department of Neurology, Galilee Medical Center, Nahariya, Israel

**Keywords:** vitiligo, Parkinson’s disease, cohort study, case–control study, association

## Abstract

**Objective:**

Our knowledge about the association between vitiligo and Parkinson’s disease (PD) is sparse. We sought to investigate the bidirectional epidemiological association between vitiligo and PD.

**Methods:**

A population-based study was conducted using Clalit Health Services (CHS) database (2002–2019) using both a cohort study and a case–control study design. Adjusted hazard ratio (HR) and odds ratio (OR) were calculated by multivariate Cox and logistic regressions, respectively.

**Results:**

Overall, 20,851 vitiligo patients and 102,475 controls were included. The incidence of new-onset PD was 2.9 (95% CI, 2.1–4.1) and 4.3 (95% CI, 3.8–4.9) cases per 10,000 person-years among patients with vitiligo and controls, respectively. Patients with vitiligo had a significantly decreased risk of developing new-onset PD [adjusted HR, 0.62; 95% confidence interval (CI), 0.43–0.89, *p* = 0.009]. On the other hand, the likelihood of having vitiligo after a preexisting diagnosis of PD was not statistically different (adjusted OR, 0.80; 95% CI, 0.61–1.06; *p* = 0.117). Relative to the remaining patients with vitiligo, those with vitiligo and comorbid PD experienced an elevated risk of all-cause mortality (adjusted HR, 2.63; 95% CI, 1.82–3.80; *p* < 0.001) and higher prevalence of cardiometabolic comorbidities.

**Conclusion:**

Vitiligo is associated with a lower risk of developing PD. The presence of comorbid PD predisposes patients with vitiligo to elevated mortality and cardiometabolic outcomes.

## Introduction

Parkinson’s disease (PD) is a neurodegenerative disorder caused by progressive degeneration of nigral neural cells of the substantia nigra of the brain stem. The estimated prevalence of PD is 0.3% in the general population, 1.0% in people older than 60 years, and 3.0% in people older than 80 years. Incidence rates of PD are estimated to range between 8 and 18 per 100,000 person-years (1). The etiology of PD is not fully elucidated, but genetic, inflammatory, and environmental factors might play a pathogenic role in the development of the disease. Autoimmunity was additionally suggested as another potential cause of this disabling neurological disease (2, 3).

Vitiligo is a chronic inflammatory disease that typically targets the skin, hair, and mucous membranes. The development of the disease is the result of the progressive destruction of melanocytes and their loss of function in specific tissues (4). This can phenotypically manifest as segmental or non-segmental depigmented patches comprising well-demarcated, non-scaly, milky-white macules (5). Vitiligo is considered the most common disease resulting in depigmentation. The worldwide prevalence is estimated to be between 0.1–2% in both adults and children. The disease affects men, women, and people from different ethnic groups and skin colors with an equal affinity (6). Intriguingly, 25% of affected individuals manifest before age 10, almost 50% will develop the disease before age 20, and nearly 70–80% before age 30 (5).

PD was evidenced to coexist with autoimmune diseases, as revealed by a recent meta-analysis that showed a 55% increased likelihood of these diseases among patients with PD^7^. The most robust of these associations refer to bullous pemphigoid, inflammatory bowel disease, Sjogren syndrome, and Grave’s disease (7). The association of PD with vitiligo, however, remains to be firmly established. The objective of the current study is to investigate the bidirectional associations between vitiligo and PD using a large-scale, population-based study. Additionally, we aimed to assess the determinants of PD among patients with vitiligo.

## Methods

### Study design and data Set

The current study aimed to investigate the bidirectional association between vitiligo and PD, a large-scale population-based study included two study designs. First, a retrospective cohort study design was used to longitudinally follow patients with vitiligo and estimate the incidence of new-onset PD. Second, a case–control study design was applied to estimate the prevalence of preceding PD (exposure) in patients with subsequent vitiligo (outcome). Considering the rare disease assumption, the latter design is likely to delineate the odds of vitiligo after PD.

We utilize the computerized data set of Clalit Health Services (CHS) as the data source. According to regulations of the National Health Insurance Law, residents in Israel are obligated to enroll in one of the four healthcare maintenance organizations. CHS is the largest healthcare provider organization in Israel, with 4,554,343 members as of 2018. CHS provides a comprehensive array of medical coverage, including both outpatient and inpatient settings. Each interaction within the healthcare system is recorded in the patient’s medical file, and the CHS database is continuously updated; the database offers an extensive overview of CHS enrollees over time, establishing its reliability as a source for epidemiological data.

### Study population and definition of covariates

The CHS database was screened for incident cases with a diagnostic code of vitiligo between the years 2002 and 2019. Patients were considered eligible for inclusion if one of the following criteria was fulfilled: (i) a documented diagnosis of vitiligo as registered by a board-certified dermatologist, or (ii) a diagnosis of vitiligo in discharge letters from dermatological wards. The diagnosis of PD was based on documentation by a certified neurologist.

We enrolled a control group including up to five individuals without vitiligo per each case. Controls were matched based on age, sex, and ethnicity and were recruited on the day in which the corresponding case was diagnosed. Outcome measures were adjusted for demographic variables and putative confounding comorbidities, including smoking, diabetes mellitus, hypertension, hyperlipidemia, and body mass index (BMI).

### Statistical analysis

The comparison of variables between different comparator groups was performed using the chi-square test and t-test for categorical and continuous variables, respectively. In the cohort study design, incidence rates of PD were calculated for both vitiligo patients and controls and expressed as the number of events per 10,000 person-years. Hazard ratios (HR) s and 95% confidence intervals (CI) s for the risk of incident PD were found using the Cox regression model. Differences in the all-cause mortality of vitiligo patients with and without PD were estimated using a stratified log-rank test.

In the case–control study design, conditional logistic regression analysis was utilized to calculate odds ratios (ORs) and 95% CIs to compare cases and controls with reference to the presence of preexisting PD. Based on the temporal relationship between exposure and outcome in case–control studies, only individuals who developed vitiligo after the diagnosis of PD were included. Two-tailed *p*-values less than 0.05 were considered statistically significant. All statistical analyses were performed using SPSS software, version 25 (SPSS, Armonk, NY: IBM Corp).

## Results

### Characteristics of the study population

A total of 123,326 participants were included in the current study. Of whom, 20,851 were patients with vitiligo, and 102,475 were age-, sex, and ethnicity-matched controls. The mean (SD) age at the diagnosis of vitiligo was 34.7 (22.4) years, 10,570 (50.7%) were females, and 15,311 (73.4%) were Jews (Table 1). The baseline characteristics of the study population are delineated in Table 1.

**Table 1:** Descriptive characteristics of the study population.

Characteristic	Patients with vitiligo (*N* = 20,851)	Controls (*N* = 102,475)	*p* value
**Age, years**			
Mean (SD)	34.7 (22.4)	34.6 (22.4)	0.608
Median (range)	32.4 (0.1–95.4)	32.4 (0.1–96.2)	
**Sex, *n* (%)**			
Male	10,281 (49.3%)	50,523 (49.3%)	0.991
Female	10,570 (50.7%)	51,952 (50.7%)	
**Ethnicity, *n* (%)**			
Jews	15,311 (73.4%)	75,249 (73.4%)	0.903
Arabs	5,540 (26.6%)	27,226 (26.6%)	
**BMI, mg/kg** ^**2**^			
Mean (SD)	25.2 (31.5)	24.8 (31.5)	0.241
**Smoking, *n* (%)**	5,207 (25.0%)	29,728 (29.0%)	<0.001
**Charlson comorbidity score, mean (SD)**	0.4 (0.9)	0.4 (1.0)	0.876

N, Number; SD, standard deviation; BMI, body mass index.

### The risk of PD in patients with vitiligo (retrospective cohort study design)

The incidence rate of PD was estimated at 2.9 (95% CI, 2.1–4.1) and 4.3 (95% CI, 3.8–4.9) cases per 10,000 person-years among patients with vitiligo and controls, respectively.

Relative to their control individuals, patients with vitiligo had a significantly decreased risk of PD (HR, 0.68; 95% CI, 0.48–0.98; *p* = 0.037). In sex- and age-stratified analyses, the reduced risk of PD was only significant among males (HR, 0.61; 95% CI, 0.38–0.98; *p* = 0.041) and older individuals (age ≥ 32.4; HR, 0.68; 95% CI, 0.47–0.97; *p* = 0.034). In a multivariate analysis adjusting for demographics and comorbidities, vitiligo was associated with a significantly reduced risk of PD (fully-adjusted HR, 0.62; 95% CI, 0.43–0.89; *p* = 0.009; Table 2).

**Table 2:** Incidence rates and hazard ratio of new-onset Parkinson’s disease among patients with vitiligo (cohort study design).

	**Vitiligo**	**Controls**
Follow-up time, PY	115,715.9	564,866.4
Median follow-up time, years (range)	5.5 (0.1–14.5)	5.5 (0.1–14.5)
Number of events	34	243
Incidence rate / 10,000 PY (95% CI)	2.9 (2.1–4.1)	4.3 (3.8–4.9)
Unadjusted HR (95% CI) [P value]	**0.68 (0.48–0.98) [0.037]**	Reference
***Sex- and age-stratified analysis***		
Male-specific HR (95% CI) [P value]	**0.61 (0.38–0.98) [0.041]**	Reference
Female-specific HR (95% CI) [P value]	0.81 (0.47–1.39) [0.440]	Reference
≥32.4 year-specific HR (95% CI) [P value]	**0.68 (0.47–0.97) [0.034]**	Reference
<32.4 year-specific HR (95% CI) [P value]	NA	Reference
***Multivariate adjusted analysis***		
Fully-adjusted (95% CI) [P value]^a^	**0.62 (0.43–0.89) [0.009]**	Reference

HR, hazard ratio; CI, confidence interval; PY, person-year; NA, not applicable.

^a^Adjusted for age, sex, ethnicity, and comorbidities.

Bold: significant value.

### The odds of vitiligo in patients with a preexisting diagnosis of PD (case–control study design)

The presence of preexisting PD was observed in 59 (0.3%) patients with vitiligo and 361 (0.4%) controls. The development of subsequent vitiligo was not significantly associated with a history of PD (OR, 0.80; 95% CI, 0.61–1.06; *p* = 0.116). In an age- and sex-stratified analysis, no significant association was noted between a history of PD and subsequent vitiligo except for the female sex group, where an inverse association was found (OR, 0.67; 95% CI, 0.45–0.99; *p* = 0.043; Table 3). In a multivariate logistic regression analysis controlling for demographic variables and comorbidities, the odds of vitiligo were statistically comparable after PD (fully-adjusted OR, 0.80; 95% CI, 0.61–1.06; *p* = 0.117; Table 3).

**Table 3:** The odds of vitiligo in patients with a preexisting diagnosis of Parkinson’s disease (case–control study design).

*N* (%) of preexisting Parkinson’s disease in patients with vitiligo	59 (0.3%)
*N* (%) of preexisting Parkinson’s disease in controls	361 (0.4%)
Unadjusted OR (95%CI) [*p* value]	0.80 (0.61–1.06) [0.116]
***Sex- and age-stratified unadjusted analysis***	
Male-specific OR (95%CI) [*P* value]	**0.67 (0.45–0.99) [0.043]**
Female-specific OR (95%CI) [*P* value]	0.98 (0.67–1.45) [0.929]
≥32.4 year old-specific OR (95%CI) [*P* value]	0.80 (0.61–1.06) [0.121]
<32.4 year old-specific OR (95%CI) [*P* value]	NA [0.524]
***Multivariate adjusted analysis***	
Fully adjusted OR (95%CI) [*P* value]^a^	0.80 (0.61–1.06) [0.117]

^*^The prevalence of Parkinson’s disease in cases when psoriasis preceded vitiligo (in cases) or preceded recruitment (in controls).

N, number; OR, odds ratio; n, number; CI, confidence interval; NA, not applicable.

Bold: significant value.

^a^Adjusted for age, sex, ethnicity, and comorbidities.

### Factors associated with comorbid PD in patients with vitiligo

The last endpoint was to characterize vitiligo patients with comorbid PD as compared to the remaining patients with vitiligo. The presence of PD in patients with vitiligo was significantly associated with older age, Jewish ethnicity, obesity, smoking, ischemic heart disease, hyperlipidemia, hypertension, and diabetes mellitus (Table 4).

**Table 4:** Determinants of Parkinson’s disease among patients with vitiligo.

	**Vitiligo with Parkinson’s disease (*n* = 93)**	**Vitiligo without Parkinson’s disease (*n* = 20,758)**	**OR (95% CI)**	***P* value**
Age at the onset of vitiligo, years; mean (SD)^a^	67.8 (11.8)	34.6 (22.3)	**2.28 (1.99–2.62)** ^**a**^	**<0.001**
Female sex, *n* (%)	46 (49.5%)	10,524 (50.7%)	0.95 (0.63–1.43)	0.812
Jewish ethnicity, *n* (%)	87 (93.5%)	15,224 (73.3%)	**5.27 (2.30–12.06)**	**<0.001**
Obesity, *n* (%)	33 (35.5%)	4,122 (19.9%)	**2.22 (1.45–3.40)**	**<0.001**
Smoking, *n* (%)	34 (36.6%)	5,173 (24.9%)	**1.74 (1.14–2.65)**	**0.010**
Ischemic heart disease, *n* (%)	30 (32.3%)	1,168 (5.6%)	**7.99 (5.15–12.39)**	**<0.001**
Hyperlipidemia, *n* (%)	71 (76.3%)	6,217 (29.9%)	**7.55 (4.68–12.19)**	**<0.001**
Hypertension	60 (64.5%)	3,298 (15.9%)	**9.63 (6.28–14.74)**	**<0.001**
Diabetes mellitus, *n* (%)	35 (37.6%)	2,296 (11.1%)	**4.85 (3.18–7.40)**	**<0.001**

n, number; SD, standard deviation.

^a^OR per 10-year increase in age.

Bold: significant values.

Survival analysis was then performed to assess the risk of all-cause mortality in patients with vitiligo and comorbid PD relative to the remaining patients with vitiligo. Comorbid PD conferred increased all-cause mortality in univariate (HR, 14.45; 95% CI, 10.04–20.79; *p* < 0.001) and multivariate (adjusted HR, 2.63; 95% CI, 1.82–3.80; *p* < 0.001) analyses (Supplementary Figure S1).

## Discussion

The current large-scale epidemiological study delineated that vitiligo confers a protective role against the development of PD, as patients with vitiligo are less susceptible to experiencing subsequent PD. The likelihood of vitiligo after PD was not statistically elevated. Relative to other patients with vitiligo, those with vitiligo and comorbid PD were at a 2.5-fold elevated risk of all-cause mortality and had a greater burden of cardiometabolic comorbidities.

A growing body of evidence suggests that PD might be associated with an autoimmune theme (8, 9). In a recent meta-analysis that pooled data from 38 studies comprising 873,643 PD patients and 13,402,821 controls, a positive association was found between PD and comorbid autoimmune diseases (7). This association might be interpreted by an immune dysfunction that may underly the pathogenesis of PD (8, 9). It has been demonstrated that an aberrant immune response may start years prior to the diagnosis of PD (10). Scientific evidence revealed that sustained inflammatory response, T-cell infiltration, and glial cell activation play a pathogenic role in the degeneration of dopaminergic neurons (9, 11).

The main autoimmune diathesis in PD refers to certain diseases, mainly bullous pemphigoid and inflammatory bowel disease (7). However, no significant association was found between PD and other prevalent autoimmune diseases, like rheumatoid arthritis, systemic lupus erythematosus, multiple sclerosis, and celiac disease (7). The association between vitiligo and PD has been sparsely investigated in the past. In a Korean nationwide retrospective cohort study, 1,500 patients with vitiligo and 13,500 controls were investigated (12). The risk of PD among patients with vitiligo was absolutely lower (1.1% vs. 1.4%, respectively), but it did not reach the level of statistical significance (adjusted HR, 0.83; 95% CI, 0.41–1.68; *p* = 0.255) (12). The results of this study align with our findings in the same direction. As our cohort encompassed a substantially larger number of vitiligo patients, we possessed greater statistical power to identify meaningful differences. The pathomechanism underlying the inverse association between vitiligo and PD remains unclear and warrants further investigation. Given that neuromelanin and melanin share some functional features and overlapping biosynthetic pathways, it has been hypothesized that genes involved in skin pigmentation and melanin formation might contribute to the susceptibility of midbrain dopaminergic neurons to neurodegeneration (13). Recent work suggests that oxidized dopamine and neuromelanin accumulation mediate the convergence of mitochondrial and lysosomal dysfunction in patient-derived neurons (14). In addition, the expression of human tyrosinase in mouse substantia nigra pars compacta led to the formation of neuromelanin, resulting in the degeneration of nigral dopaminergic neurons (14). Based on the aforementioned evidence, the fact that neuromelanin accumulation might trigger degeneration of dopaminergic neurons might explains, at least in part, the inverse association of vitiligo and PD. As the levels and/or function of neuromelanin are expected to be lower among patients with vitiligo, this might confer a sort of protection against the development of PD. This explanation remains hypothetical, and further basic research is necessary to establish or refute it.

The elevated risk of death among patients with vitiligo and comorbid PD, relative to those with isolated vitiligo, is conceivable considering the known fact that PD itself carries an elevated risk of mortality (15). Additionally, we found that patients with a dual diagnosis of vitiligo and PD were found to experience a higher burden of cardiometabolic conditions. This accords with the elevated risk of cardiovascular and metabolic outcomes associated with the presence of PD (16–19).

The current large-scale study throws a spotlight on a poorly investigated topic and might clarify some uncertainties that surround the burden of PD in vitiligo. The large study population and ability to obtain all relevant medical information from a centralized source represent the major strengths of our study. Moreover, vitiligo and PD diagnoses were all made by board-certified dermatologists and neurologists, respectively. This corroborates the validity of the diagnosis and argues against the presence of meaningful misclassification. Our study, however, is limited by the absence of data on the extent, duration, activity, and distribution of vitiligo and the severity of PD. The relatively homogenous ethnic background of eligible patients embodies another limitation of the current study. Therefore, it is important to explore this topic in other study populations originating from different ethnic backgrounds. Other limitations stem from a lack of data about vitiligo-related treatments and their influence on the risk of PD and the lack of data about other autoimmune comorbidities.

In conclusion, the current large-scale study demonstrated that Israeli patients with vitiligo have a decreased risk of subsequent PD. Vitiligo patients with comorbid PD are at increased risk of all-cause mortality and cardiometabolic conditions as compared to vitiligo patients without these comorbidities. Additional studies investigating this association in other study populations originating from different ethnic backgrounds are necessary.

## Data availability statement

The raw data supporting the conclusions of this article will be made available by the authors, without undue reservation.

## Ethics statement

Ethical review and approval was not required for the study on human participants in accordance with the local legislation and institutional requirements. Written informed consent from the patients/participants or patients/participants’ legal guardian/next of kin was not required to participate in this study in accordance with the national legislation and the institutional requirements.

## Author contributions

KK: Conceptualization, Formal analysis, Investigation, Methodology, Resources, Writing – original draft, Writing – review & editing. LO: Methodology, Writing – original draft, Writing – review & editing. OW: Validation, Writing – original draft, Writing – review & editing, Investigation, Project administration, Software. SB: Resources, Supervision, Validation, Writing – original draft, Writing – review & editing.
