# Supplementary figures and images for: Clarifying the association between Parkinson’s disease and vitiligo: a population-based large-scale study
Source: Front Neurol. 2024 May 21;15:1387404. doi: 10.3389/fneur.2024.1387404 (PMC11148865; doi:10.3389/fneur.2024.1387404)

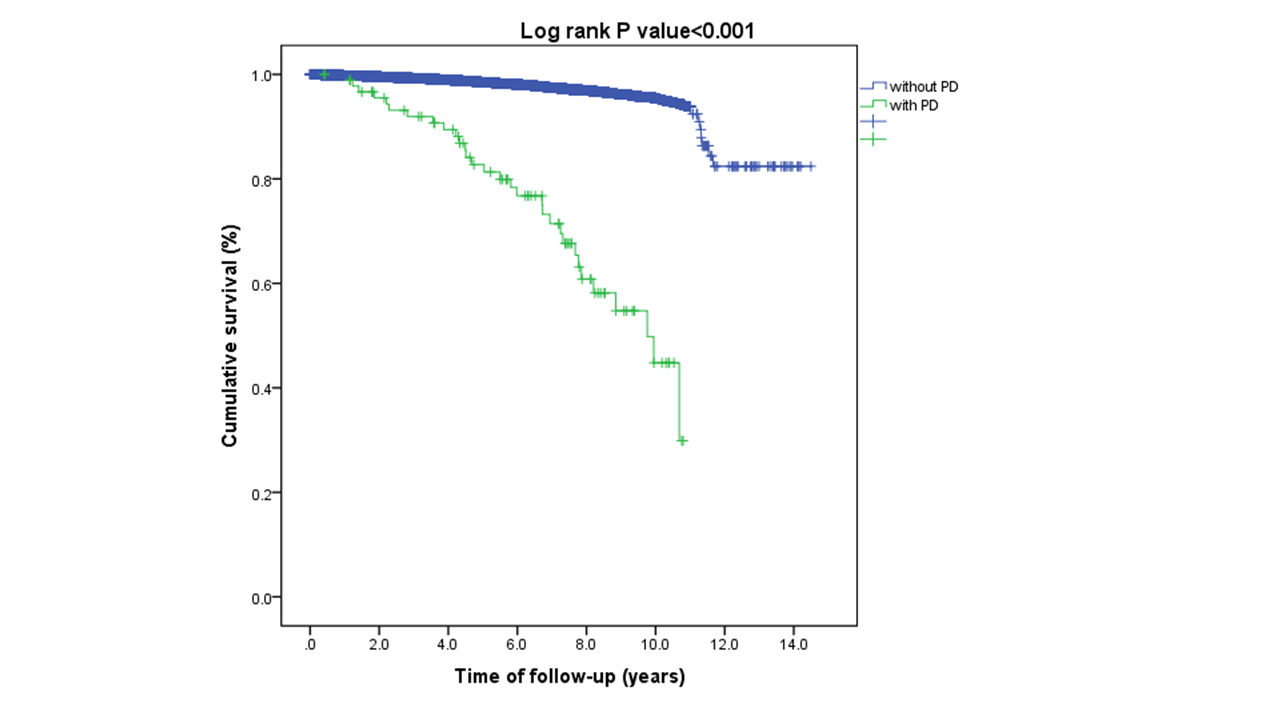

Supplement: Supplementary file 1 [file Image_1.tif]
